# Will it run?—A proof of concept for smoke testing decentralized data analytics experiments

**DOI:** 10.3389/fmed.2023.1305415

**Published:** 2024-01-08

**Authors:** Sascha Welten, Sven Weber, Adrian Holt, Oya Beyan, Stefan Decker

**Affiliations:** ^1^Chair of Computer Science 5, Rheinisch-Westfälische Technische Hochschule (RWTH) Aachen University, Aachen, Germany; ^2^Institute for Biomedical Informatics, Faculty of Medicine and University Hospital Cologne, University of Cologne, Cologne, Germany; ^3^Fraunhofer Institute for Applied Information Technology FIT, St. Augustin, Germany

**Keywords:** decentralized applications, federated learning, machine learning, software testing, simulation, web services

## Abstract

The growing interest in data-driven medicine, in conjunction with the formation of initiatives such as the European Health Data Space (EHDS) has demonstrated the need for methodologies that are capable of facilitating privacy-preserving data analysis. Distributed Analytics (DA) as an enabler for privacy-preserving analysis across multiple data sources has shown its potential to support data-intensive research. However, the application of DA creates new challenges stemming from its distributed nature, such as identifying single points of failure (SPOFs) in DA tasks before their actual execution. Failing to detect such SPOFs can, for example, result in improper termination of the DA code, necessitating additional efforts from multiple stakeholders to resolve the malfunctions. Moreover, these malfunctions disrupt the seamless conduct of DA and entail several crucial consequences, including technical obstacles to resolve the issues, potential delays in research outcomes, and increased costs. In this study, we address this challenge by introducing a concept based on a method called Smoke Testing, an initial and foundational test run to ensure the operability of the analysis code. We review existing DA platforms and systematically extract six specific Smoke Testing criteria for DA applications. With these criteria in mind, we create an interactive environment called Development Environment for AuTomated and Holistic Smoke Testing of Analysis-Runs (DEATHSTAR), which allows researchers to perform Smoke Tests on their DA experiments. We conduct a user-study with 29 participants to assess our environment and additionally apply it to three real use cases. The results of our evaluation validate its effectiveness, revealing that 96.6% of the analyses created and (Smoke) tested by participants using our approach successfully terminated without any errors. Thus, by incorporating Smoke Testing as a fundamental method, our approach helps identify potential malfunctions early in the development process, ensuring smoother data-driven research within the scope of DA. Through its flexibility and adaptability to diverse real use cases, our solution enables more robust and efficient development of DA experiments, which contributes to their reliability.

## 1 Introduction

Data-driven analyses, such as basic statistics or Machine Learning (ML)-based approaches, have been extensively used for analyzing data in a variety of applications such as medical diagnosis and treatment or financial business intelligence (1–3). Traditionally, data is collected from several sources, stored in a central location, and analyzed by scientists. However, data centralization poses several challenges (4). For example, due to the exponential growth of data, the gathered data volume might not allow central storage, or in some cases, it would be too expensive (5). Besides these technical challenges, regulations such as the General Data Protection Regulation (GDPR) in the European Union^1^ prohibit or limit the centralization of personal data due to privacy concerns and its level of sensitivity. This issue is particularly present in domains such as healthcare, where personal data is protected (5). In the context of the European Health Data Space (EHDS)^2^, the issue of accessing fragmented and silo-ed data is intended to be resolved through the implementation of Federated Health Data Networks (FHDNs) that consist of decentralized and interconnected nodes, allowing data to be analyzed by participants of the FHDNs (6). In order to enable data analysis across multiple nodes, key technologies for DA [such as Federated Learning (FL)] have been considered as indispensable and proposed as a solution by omitting the need for data centralization (7, 8). Here, the analysis code is executed at the data source(s), and only the (intermediate) analysis results, such as aggregated statistics or, in ML-terms, model parameters, are transmitted between the data providers rather than sharing actual data instances. DA provides solutions for several legal considerations such as patient data ownership or data control (9). This includes compliance with measures such as the GDPR. Furthermore, ensuring transparent and accountable access to this data is crucial to uphold privacy and security standards (9). Since it addresses challenges, such as data privacy, high storage costs, or long transfer times, Distributed Analytics (DA) has recently gained attention and has found applications in various use cases, including skin cancer classification, predictive modeling using radiomics for lung cancer, brain tumor segmentation, and breast cancer detection (5, 10–14).

**Figure 1:**
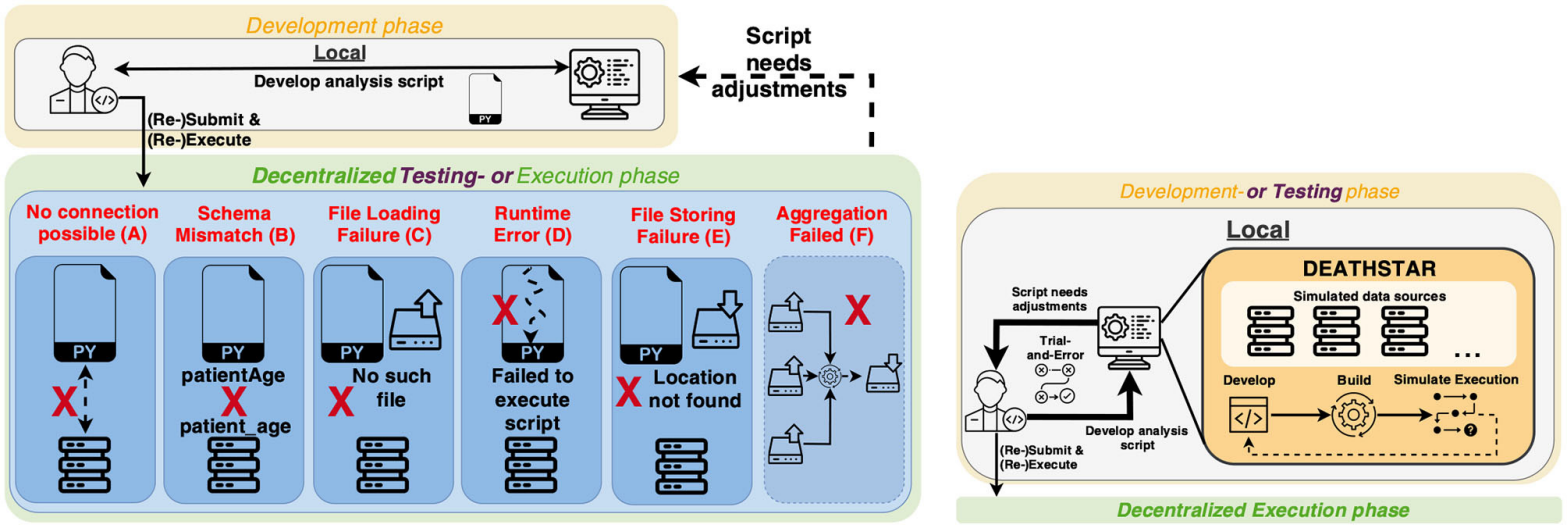
Proposed concept for local smoke tests. Up to now, the (smoke) testing phase of the analysis code has been postponed to the actual execution, making it difficult to differentiate between the testing and execution phases **(left)**. As a result, any code errors can only be identified by running the code at each data provider. This dependence on distributed data providers can make creating DA code time-consuming and cumbersome. Instead of smoke testing during the execution phase itself, we propose DEATHSTAR to iteratively trial-and-error the analysis script locally **(right)**.

Before analyses can deliver their full potential, several steps must be taken to build an error-free and robust analysis code. Among other steps, we recognize three essential phases: Development, testing, and execution phase (Figure 1) (15). The development phase involves implementing the code, covering a data pre-processing routine and the analysis script. During the testing phase, there may be two types of testing scenarios: one is testing from a software perspective that ensures the code is executable. The other is analysis validation using test data to assess performance. The execution phase covers the application of the analyses on real data to obtain actual analysis outcomes. At this point, it becomes evident that these standard workflows assign an essential role to the availability of data: Without sufficient data, fast prototyping through, e.g., trial-and-error and software tests, can be only conducted on a limited basis. Moreover, up to now and to the best of our knowledge, how DA code is tested has been left to the developer's responsibility and intuition, showcasing a lack of clearly defined testing criteria and capabilities in the domain of DA. This circumstance entails a specific degree of uncertainty regarding the analysis code during its execution: Will it run? The consequence is that insufficiently tested analysis code is susceptible to single points of failure (SPOFs) during the execution phase, such that another development round is needed to fix the code (Figure 1). Due to the decentralized nature of DA, any kind of errors during the execution require the analysis code to be re-built, re-distributed to the data holders, and re-executed (Figure 1). This re-distribution is time-consuming and potentially involves multiple parties, e.g., in the medical domain, where the analysis has to be verified before interacting with data. Thus, there is a need for adequate testing criteria and capabilities that identify potential malfunctions in the code before its execution.

### 1.1 Objectives

To establish an initial foundation for testing in DA, we derive requirements for DA code, which should be fulfilled to ensure that the analysis code is operational. We aim to define criteria for a testing approach called *Smoke Testing* to support developers in their development process (16, 17). These criteria constitute the minimum requirements for DA code that must be guaranteed before its execution. We hypothesize that without these requirements the DA code will definitely fail or cause undesired behavior. In summary, we evaluate the following research question:

**RQ1** What are suitable Smoke Testing criteria for DA executions?

Secondly, we intend to develop a Smoke Testing suite as a Proof of Concept (PoC), specifically designed to evaluate analysis code according to our defined criteria. Since data is essential to test data-driven analysis code properly, we aim for a Smoke Testing suite capable of generating data instances that can be used for Smoke Testing, making our approach less reliant on prior data-sharing. Regarding this, we hypothesize that a simulation-based Smoke Testing suite reduces the dependence on data providers. One of our core assumptions is that data schema details are shared, while actual sensitive data instances can be kept under seal by the data providers. To reach this goal, we will evaluate the following research questions:

**RQ2** What is necessary to enable Smoke Testing on DA code?
  **RQ2.1** How can privacy-preserving testing of DA algorithms be enabled?
  **RQ2.2** How can the execution of DA algorithms be (Smoke) tested without a real DA environment?

### 1.2 Contributions and findings

Aligned with our objectives from the previous section, this work presents the following contributions:

- We propose six criteria for Smoke Testing that we derive from a literature review of DA infrastructure implementations. Those criteria must be met by DA analyses in order to ensure their operability.
- We developed and implemented a Smoke Testing suite, called Development Environment for AuTomated and Holistic Smoke Testing of Analysis-Runs (DEATHSTAR)^3^. DEATHSTAR employs a *testing-through-simulation* approach to identify potential malfunctions in the analysis code by systematically validating our six criteria. This PoC, inspired by Integrated Development Environments (IDEs), allows the prototyping and simulation of DA experiments on synthetic or (real) sample data.
- We conduct a User-Study with 29 participants to evaluate the effectiveness of our criteria and the usability of DEATHSTAR.
- We lastly present a technical evaluation demonstrating the flexibility and adaptability of our approach by successfully repeating and reproducing three real-world use cases.

Overall, we find that almost all DA algorithms (96.6%), developed and (Smoke) tested by participants of our User-Study using our approach, terminated with no errors in a real DA execution. These results suggest that the six criteria we proposed are sufficient for ensuring the operability of the analysis code. Additionally, we achieved a System Usability Scale (SUS) score of 88.3 in our User-Study, which is considered to be “excellent” (18). The outcomes of the second part of our evaluation show that our concept can support DA-driven research under real circumstances and is flexible enough to serve various data types and sources.

## 2 Method

In the previous section, it became apparent that the essential element of DA approaches is the analysis code. As these analyses are executable software fragments, they can consequently be vulnerable to unexpected failure during the execution, like any software product (19). For example, the algorithm might not be compatible with a specific data source version or contain a logical error that needs to be resolved before the execution (see Figure 1). As the most widespread method to verify software quality, testing can prevent such failures (19). Moreover, the importance of testing is also evident when reviewing so-called Software Development Life Cycles (SDLCs) (20). These SDLC models describe systematic processes on how software should be developed and what steps should be taken in the SDLC (21). Consequently, an SDLC model can control costs, reliability, performance, and functionality of the developed software (21). As a result, various SDLC models have been developed and play a significant role in software engineering (15). It is worth noting that each SDLC model embraces a testing phase, which emphasizes that testing is indispensable in professional software development (15). Specifically for DA, the necessity of testing capabilities has already been formulated in work by Bonawitz et al. who state that an environment for testing and simulation of analysis algorithms is a requirement for DA platforms (22). One specific testing method playing a major role in this work is called *Smoke Testing* (16, 23, 24). This term stems from the industry and includes an initial and fundamental test run to ensure that a program—here: the analysis—is operational, executes successfully, and does not *end up in smoke*. For example, Herbold and Haar successfully applied Smoke Testing to find problems in analytics software libraries and algorithms (16). Specifically, they designed a total of 37 Smoke Tests for classification- and clustering algorithms (16).

The methodology of this paper is inspired by the work of Cannavacciuolo and Mariani (17), who applied Smoke Testing to cloud systems, intending to validate whether a system is operational post-deployment, which helped to determine if more sophisticated tests can be conducted. As part of their work, they propose several Smoke Testing criteria that can be used as a foundation for creating Smoke Testing suites in the scope of cloud systems (17). Since the relevant DA platforms discussed in Section 2.2 are typically not deployed in cloud systems, and our primary emphasis is on (Smoke) testing analysis code rather than an entire infrastructure, these criteria are not applicable to our specific scenario. Nevertheless, they have specified three key characteristics of Smoke Tests, that serve as an inspiration for our work. Those characteristics define the way *how* Smoke Testing criteria should be validated:

- **Shallow:** Smoke Tests should be kept at a higher abstraction level and not overly detailed. This means that only a system's or software's basic functionality and operability should be validated. It is just serving as a prerequisite for more sophisticated testing methods.
- **Fast:** Smoke Tests must be fast in their execution since they are performed before other test runs or, in our scenario, the analysis execution.
- **Automatic:** As an extension to the *fast* characteristic, Smoke Tests should be fully automated to reduce manual intervention.

To realize Smoke Testing suites, so-called playgrounds or prototyping environments may provide a possible solution (22). Here, the term playground refers to services that allow users to interact and *play* with software without prior complex setup or configuration (25). Moreover, these playgrounds enable users to iteratively (i.e., trial-and-error) develop and priorly test their entire implementation or specific modules (25, 26). Because playgrounds have successfully enabled testing approaches in other settings, our work pursues a similar approach (25–28).

We begin the conceptualization of such a Smoke Testing suite by abstracting and formalizing the scenario, focusing on the relevant steps in which the analysis execution might fail based on related works in the DA domain (Section 2.2). Moreover, our approach aims for a user-centric design, so we initially describe the problem statement from a user perspective (Section 2.1). The outcome of this abstraction is a formal model that describes the analysis process of the code, which is distributed within a DA infrastructure. Based on the steps in the process model, we derive our set of Smoke Test criteria that aim to ensure that each step can be executed (Section 2.3). We aim to keep the set of criteria as “shallow” as possible to comply with the defined characteristics of Smoke Testing (see above). Subsequently, we present a PoC implementation that can apply Smoke Tests to analysis code based on our defined criteria (Section 2.4). We aim for a “fast” and “automated” solution consistent with the Smoke Testing characteristics. Lastly, we evaluate the effectiveness of our solution, its usability, and we apply it to three distinct use cases as part of our technical evaluation (Section 3). For the implementation and evaluation, we use the DA platform PADME as infrastructure to execute the analyses (7).

### 2.1 User-centered problem description

Initially, developers or scientists who intend to conduct a DA experiment need to develop the code for the analysis, which is designed to analyze data provided by decentralized data holders (see Figure 1). The development process usually occurs locally or on a machine the developer can access. It is vital to test the analysis code to ensure its proper operation after the development (or even during it, through a trial-and-error approach). While certain parts and components of the code can be tested on a module-by-module basis, the presented setting has a shortcoming: To conduct a complete test of the code, the developer requires (sample) data to execute the developed algorithms on. However, the availability of sufficient and potentially sensitive data for testing purposes is not guaranteed due to the mentioned data protection and privacy regulations. As a result, researchers are left with two options. In case sample data is available, following an *ad hoc* testing approach might not cover all criteria that are needed to ensure the operability of the code. Secondly, in the worst case, the developer is obliged to submit the analysis script to each data provider and wait for its execution on their data in order to identify potential issues in the code. These circumstances result in an inefficient development process since the developer is reliant on the data providers, and even minor malfunctions (such as Index-out-of-Bounds, Nullpointer, TypeCast exceptions) can cause a new development round. From an abstract perspective on this scenario, the testing phase is closely coupled with the actual execution phase, which causes the mentioned inefficiency (see Figure 1). Usually, the testing phase is designed to support the development phase to allow for fast code updates and trial-and-error development. Therefore, in this work, we aim to separate the testing and execution phases and provide a solution that facilitates Smoke Testing during or after the development phase (see Figure 1, right).

### 2.2 Abstract workflow

Our initial step involves examining how the analysis code operates on a conceptual and abstract level. In general, two execution policies exist that enable DA: A parallel and a sequential approach (sometimes referred to as FL and Institutional Incremental Learning (IIL), respectively) (13, 29). In IIL, the data holders are arranged in a sequence, and the analysis code is sent from institution to institution until the last institution sends the final (and aggregated) results back. The procedure for FL repeats the following steps: First, the analysis algorithm is simultaneously distributed to all participating data holders. Then, each data holder executes the analysis algorithm on the local data and sends the result of this analysis back to the central component. The central component aggregates all partial results, combining the results of all participants. This aggregated result is either the final or intermediate result for the next so-called communication or federated round. The conduct of a DA experiment generally requires an infrastructure that orchestrates the analysis and transmits the code to the data holder according to one of the foundational execution policies mentioned above. In recent years, several implementations of DA have been proposed. DataSHIELD (DS) is an open-source solution that follows the FL approach and uses the programming language *R*, often used in statistics^4^ (30, 31). Another emerging concept is the Personal Health Train (PHT), which follows the sequential paradigm. The PHT uses software containers^5^ to distribute the analysis code to each data provider. Some implementations following the PHT concept are Vantage6, PHT-meDIC, and PADME by Welten et al. (7, 32, 33). Besides FL and IIL, additional (hybrid) approaches for DA exist: Swarm Learning (SL) and Secure Multiparty Computation (SMPC), which use Peer-To-Peer (P2P) communication instead of relying on a central component (34, 35). These infrastructures, founded on the dispatching paradigms, such as IIL and FL, serve as the source for our abstraction.

**Figure 2:**
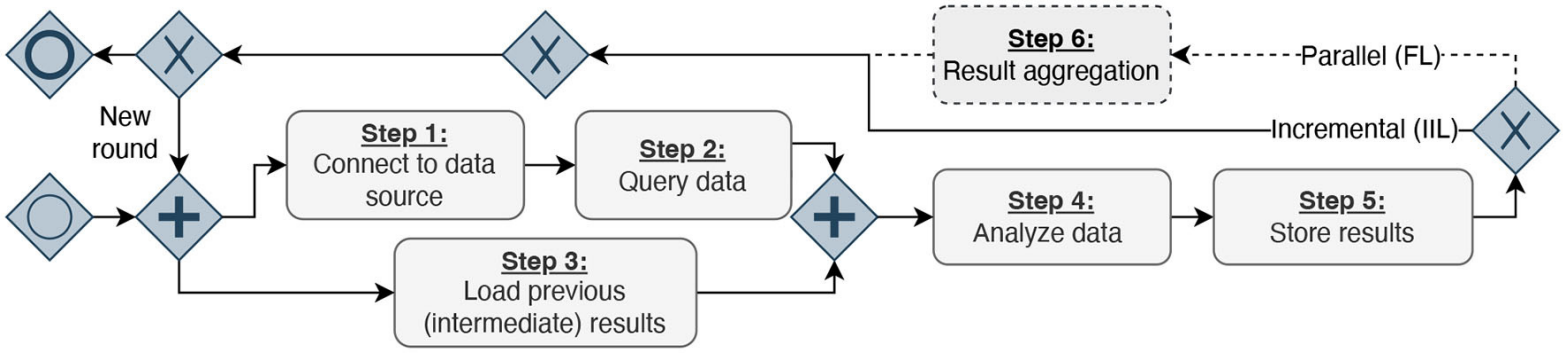
Process diagram inspired by the business process model and notation, displaying the identified six steps performed in DA experiments. First, the analysis code needs to connect to a data source and query analysis data (Steps 1 & 2). Simultaneously, the code can load results from previous executions or initial models and weights (Step 3). Afterward, the analysis is executed, and the results are stored (Steps 4 & 5). The results must be aggregated depending on the DA architecture (Step 6). Finally, either a new execution round is triggered, or the execution finishes.

After systematically reviewing these infrastructures, studies conducted with them, and our personal experiences from DA experiments, we have identified six abstract steps (S1–S6) that the analysis code performs during its execution, as shown in Table 1. We transformed our findings into a process diagram for a better overview of the abstract workflow (Figure 2). Despite how the (intermediate) results are finally combined, the infrastructures do not differ in their workflow on the conceptual level. First, the developed code must establish a database connection (S1). Then, the analysis queries the data (S2) and loads the intermediate results (S3) from previous execution rounds. The queried data from Step 2 and the previous results from Step 3 serve as the input for a generic analysis code. During the data analysis (S4), the queried data is used to compute updated analysis results. Once the analysis terminates, the updated results are stored (S5). In the IIL-setting, the results are stored in the analysis payload, which is then transmitted to the next data provider. In contrast, for FL, the results are directly transmitted to a central aggregation component, where the intermediate results of all analysis replicas are aggregated into a single global result (S6). As each approach we examined is round-based, these six steps are repeated in each subsequent round. In the IIL scenario, a new round starts after the analysis has been sent to the next data holder. On the other hand, in the FL scenario, a round begins after the aggregator has combined all results. Hence, the approaches following the paradigm of parallel analysis executions undergo an additional step.

**Table 1:** Applicability of the six steps identified in this paper to different DA infrastructures.

**References**	**S1**	**S2**	**S3**	**S4**	**S5**	**S6**
PHT (IIL) (7, 32, 33)	✓	✓	✓	✓	✓	
DS (FL) (31)	✓	✓	✓	✓	✓	✓
Swarm Learning (P2P) (35)	✓	✓	✓	✓	✓	✓
SMPC (P2P) (36)	✓	✓	✓	✓	✓	(✓)

Steps required by an infrastructure (row) are shown as checkmarks in the respective column. All infrastructures require connecting to a data source (S1), querying data (S2), loading previous results (S3), executing the analysis (S4), and storing results (S5). Some infrastructures require result aggregation (S6).

### 2.3 Criteria definition

Now that we have our abstract workflow model, we define six criteria that must be fulfilled to ensure that the analysis code is operational in every of our derived execution steps. For each requirement, we linked the corresponding step in our workflow.

**Requirement A: Proper connection interface**. The analysis code should be able to establish a connection to the data source without any issues. This necessitates that the algorithm's configuration is compatible with the data source's connection interface(s). Proper configuration implies that all connection parameters (e.g., file path, hostname, port number, or database type) are correct and available (S1).

**Requirement B: Matching schema**. The analysis code should be able to send syntactically correct queries to the data store and receive corresponding results in response. Hence, the expected data schema of the analysis code must match the actual data schema of the data source. Note that Requirement A focuses on the technical aspect of connecting to the data source. Requirement B refers to successfully establishing a connection based on data (schema) compatibility (S2).

**Requirement C: Load previous (intermediate) results**. Loading the (intermediate) results from previous executions into the analysis code is necessary to enable result updates, representing the core functionality of DA. In the first round, we require a successful initialization if necessary (S3).

**Requirement D: Analysis execution without errors**. If the Requirements **A**, **B**, and **C** hold, the actual DA algorithm should run without encountering any errors. An error-free execution is indicated by, e.g., the exit code 0 (S4).

**Requirement E: Successful result storage**. The analysis code should save the analysis results in the appropriate location and format. The term “correct location” refers to emitting the results as either a file or a processable bit string for transmission. This guarantees extractable analysis results, which the researcher can inspect after the execution (S5).

**Requirement F: Successful result aggregation**. In aggregation-based approaches (e.g., FL), we additionally require that the central aggregation of the intermediate results computed and stored in steps 4&5 terminates without an error (S6).

It is worth noting that we interpret these six requirements as the root causes of SPOFs and as the fundamental factors that must be met for an analysis to terminate properly. As such, these requirements only represent a subset (see “shallow” criterion) of potential additional criteria. To illustrate, it may be necessary to ensure a reliable and low-latency connection between the entities involved in DA to guarantee the proper transmission of the analysis code. However, we argue that such criteria are mainly subject to the responsibility of the DA infrastructure providers rather than the developers of the analysis code. Consequently, we have only considered requirements that developers and the analysis codes can directly influence. Additionally, we do not check for the plausibility of the results. Since DA can cover a wide spectrum of analysis types, we argue that validating the result's plausibility might contradict the “shallow” and the “fast” criteria since possible tests might be too detailed in our DA setting. For example, Smoke Tests for classification and clustering algorithms have already been proposed by Herbold and Haar (16).

### 2.4 Implementation of DEATHSTAR

**Figure 3:**
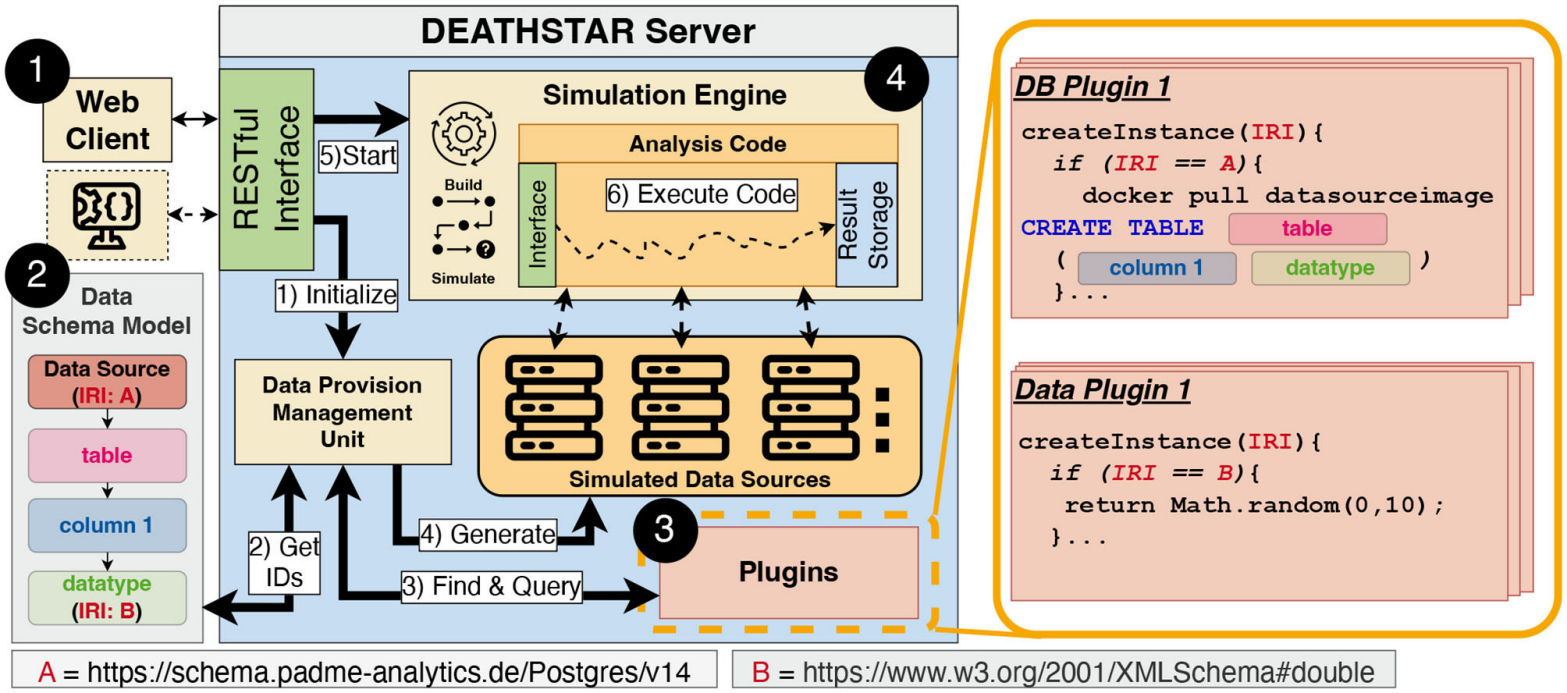
Overview of the DEATHSTAR architecture, containing a web application following the client-server paradigm. The server is a monolithic application that implements each sub-component as a library. Moreover, the overview depicts the process of generating simulated data sources. This process leverages Internationalized Resource Identifier (IRI) to find and query plugins that generate synthetic data and the data source instances. After the simulated data sources have been setup, the Simulation Engine ➍ simulates a distributed execution of the provided code.

With the foundations established in the previous section, we proceed to our PoC implementation that we refer to as DEATHSTAR. This prototype evaluates the analysis code as per our six criteria. According to the key characteristics of Smoke Testing, DEATHSTAR should offer capabilities for “fast” and “automated” Smoke Testing. To accomplish this, we adopt a *testing-through-simulation* approach, which simulates an entire DA execution with multiple rounds and data sources to detect possible non-compliances with our six criteria. Beyond this aspect of fast test automation, we also focus on a user-centric design that is inspired by IDEs and playgrounds as common tools in software engineering. To provide an overview, we have provided a top-level architectural diagram in Figure 3.

We developed a containerized web application in Node.js, using the client-server paradigm (see Figure 3), which enables the integration into other ecosystems via the provided API (component ➊). Through the use of containerization this application can be run platform independent. Moreover, the provided API can also be used in CI/CD pipelines and other IDEs, enabling developers to integrate the functionalities of DEATHSTAR into broader development processes. The User Interface (UI) includes elements that support developers in writing code and monitoring the simulations via log outputs. Our implementation is accessible under the MIT license via the repository associated with this paper. This repository offers technical descriptions, screenshots, and a video demonstrating the described features. The following sections provide a more detailed description of the architectural design.

#### 2.4.1 Data schema model ➋

We assume that the developer has access to the data schema information and the technical details of the data sources. In this work, we intend to replicate the real data sources of a DA infrastructure for our simulation and fill each replicated data source with synthetic data following the same schema and format. As no real data is involved, we claim that this approach is privacy-preserving and satisfies our aforementioned objectives. It should be noted that we consider the term *real* to be associated with sensitive and non-shareable data. In some instances, such as data donations, the developer may have access to *real* sample data, which can be used for our Smoke Testing scenario. In the latter case, we also demonstrate that our targeted approach can handle *real* sample data beyond the synthetic data we generate. Describing the structure of data sources used for data analysis is challenging because of the sheer amount of data storage technologies, data types, and their combinations. For these reasons, our goal is to find a solution that can enable the initialization of the database, the management and creation of the data structure, and the insertion of synthetic data while allowing extensions to support different data sources and data structures in the future.

A common way of specifying data structures and data formats are Data Schema Models (37, 38). We have decided to use the widely used and well-established Resource Description Framework (RDF) and its serialization Turtle^6^ (39). RDF is very flexible regarding extendability, adaptability, and granularity level. By utilizing RDF, we can model the hierarchical fashion of data sources (see Figure 3), starting from the database technology, via the inlying tables to the atomic data types of attributes. Moreover, RDF's graph-based nature enables us to model more complex data structures with interconnections between data entities by additional arcs and nodes added to the graph. Further, we used RDF in conjunction with the Web Ontology Language (OWL) to model and represent data structures, making it a versatile tool that facilitates interoperability and reusability on data-level^7^. An integral part of RDF are IRIs, which uniquely identify the entities described in the RDF model. In our case, this means that data sources or atomic data types are represented by an IRI. Two example IRIs are depicted in the Data Schema Model in Figure 3. IRI A represents the identifier for a specific data source technology, whereas IRI B refers to the atomic data type double. For the sake of simplicity, Figure 3 only shows the model for one specific data source, i.e., a data provider. To represent multiple data providers, which might participate in a DA execution, additional Data Schema Models in the same format can be added. The Data Schema Model is usually specific for one DA use case involving multiple data sources. Therefore, it is mandatory to initially model each data provider manually or with semi-automated means. While our schema as mentioned above only models the structure of the data source, we further need a mechanism to instantiate actual data sources and generate data.

#### 2.4.2 Plugin system ➌

We decided to leverage a module-based plugin system with standardized interfaces to handle the instantiation and generation of multiple data sources and synthetic data (see Figure 3). There are two general types of plugins: The first type, called Database Plugins (DB Plugins), manages the data sources (e.g., PostgreSQL) and their underlying structures (e.g., tables and columns). The second type, the Data Plugins, produces new data instances of a specific data type. Both types of plugins are available and provided as Node.js modules within the DEATHSTAR server and loaded when the application starts. Therefore, the benefits of using IRIs have become apparent at this point: Each modeled data source and type is linked to exactly one instantiation function of a plugin via an IRI.

Consequently, we can explicitly define how to instantiate a data source or generate a data instance. Developers can leverage the flexible plugin system to establish databases according to the “mix-and-match” principle, allowing them to combine complementary data plugins to populate the database. Our collection of 30 plugins for the most common atomic data types are available open-source^8^ for reuse or can be used as templates for the development of new plugins.

To manage the various types of storage technology, we rely on software containers, more specifically Docker containers^9^, to create a new instance of a data source through our DB plugins mentioned above. This approach allows us, for example, to instantiate a separate container for each required data source using a single Docker API call. Moreover, most data sources like PostgreSQL, MongoDB, MinIO, or Opal already provide images of various versions for the Docker environment that can be used as a starting point. Further, containers provide standardized connection interfaces, which facilitate the insertion of data instances into the database. We argue that this approach is versatile enough to support highly-customized storage technologies since containers can also be pulled from private repositories. Additionally, developers are also able to use *real* data samples with DEATHSTAR by using a custom plugin that either provides a proxy for the connection to an already existing data source or creates a data source that uses the *real* data samples instead of the generated ones.

#### 2.4.3 Simulation engine ➍

The task of the Simulation Engine is to take analysis code and simulate a DA execution on the data sources, which have been introduced in the previous sections. At this point, we face another challenge regarding the analysis code that could range from basic statistics to even complex code for ML model training, including a data-preprocessing pipeline, and can be written in different programming languages. Hence, our solution must be independent of the analysis complexity and the technology stack used. In order to achieve this goal, we make use of the containerization technology again and containerize the analysis code before the actual simulation. This means that the developer has all the necessary degrees of freedom to develop the analysis code with DEATHSTAR. For example, our concept is compatible with all widely used ML frameworks such as PyTorch^10^ or Scikit-Learn^11^. Apart from the analysis code, we only need the image building file (e.g., Dockerfile), which gives the instructions for building the container. To simplify this process, we offer Dockerfile templates for the most popular programming languages used in data science, such as Python^12^ and R^13^.

We chose to implement the IIL and FL paradigm in our Simulation Engine, giving us one representative of DA approaches with and without aggregation. Moreover, we argue that the implementation can be extended, if needed. For the simulation of the IIL paradigm, the developer has to provide the mentioned Dockerfile and the analysis code. In the FL scenario, we additionally require code for the aggregation component. The Simulation Engine manages the simulation process, which builds the analysis container(s). The simulation proceeds as follows: Upon building the analysis container, the engine injects DB-plugin-provided connection credentials through environment variables into the container. It then launches the analysis container, which executes the analysis code. It should be noted that in FL, these preliminary steps may occur simultaneously for each replica of an analysis container. The analysis itself adheres to the abstract workflow presented in Figure 2. It takes the received credentials and establishes a connection to the simulated data source (S1). The analysis code queries the data (S2), loads previous results if available from the filesystem of the analysis container (S3), processes, and analyzes the queried data (S4). The computed analysis results are saved in the container, which is then stopped by the Simulation Engine. A new container is instantiated from the stopped container, which carries out steps S1–S5 using the previous results and the next data source. This represents the transfer from one data source to the next, enabling us to simulate the IIL paradigm. On the other hand, in the FL case, the engine initiates a container containing the aggregator code, which has to be provided by the developer. This container gets the intermediate results produced by each replicated analysis container from the Simulation Engine, which extracts them from a pre-defined path. The aggregation container then combines the provided intermediate results into a single global result (S6) before a new analysis round begins. It is important to mention that each data source is simulated within its own virtual network. This approach prevents any side effects, like duplicated hostnames between institutions, and ensures the simulation accurately reflects the real execution environment. Moreover, using virtual networks, the Simulation Engine can be adjusted for the FL case to exchange intermediate results trough the network.

## 3 Results

In order to evaluate our Smoke Testing approach, we divided our evaluation into two parts to assess different aspects of our concept. First, we invited potential users and conducted a User-Study with an accompanying survey (Section 3.1). Through this User-Study, we investigate the effectiveness of our criteria. Secondly, as part of a technical evaluation, we replicate several real-world use cases to evaluate the fitness of our realization *in operando* (Section 3.2)^14^.

### 3.1 Evaluation of the effectiveness

**Figure 4:**
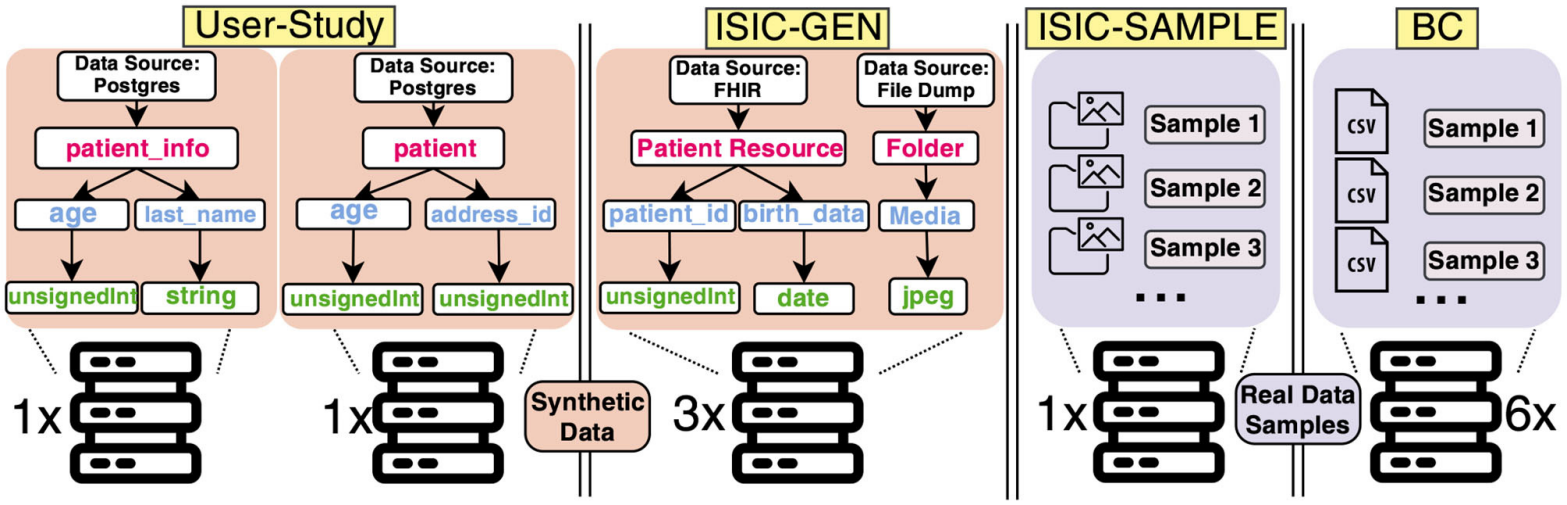
Our evaluation involved RDF data structures in the user-study and three application scenarios: International Skin Image Collaboration (ISIC)-GEN, ISIC-SAMPLE, and the Breast Cancer (BC) use case. The user-study used two distributed data sources with synthetic data, while ISIC-GEN used three data sources with synthetic data, and ISIC-SAMPLE used one data source with real sample data (13). Lastly, the BC use case leveraged six data sources with real sample data (14).

This part of our evaluation has two goals. Firstly, we want to determine the effectiveness of our defined criteria through DEATHSTAR by conducting an exemplary DA use case (called User-Study, see Figure 4). Besides this, we want to assess the contribution of our concept to the development phase of DA experiments from a user perspective and surveyed the users after their development. It should be noted that the scope of this User-Study is limited to the development of a basic statistical query rather than a complex ML model. This is due to the potential difficulty and complexity of conducting a User-Study for the latter. However, we argue that the six criteria established in this study remain relevant and applicable, regardless of the level of complexity involved in the analysis, or more specifically, in S4 (Figure 2). In either scenario, data must be queried and processed, and the results must be stored.

#### 3.1.1 Setup

We designed an exemplary use case that might occur in a real clinical study^15^. The use case aims to determine the number of patients in two hospitals that are at least (≥) 50 years old. Since we assume that these two hospitals, i.e., data providers, exist in our real ecosystem, we consequently need to re-model these, called *Hospital A* and *Hospital B*, with DEATHSTAR. Both offer a relational PostgreSQL database that provides patient information. The database at *Hospital A* contains data on patients and their treatment history, while *Hospital B* provides data on patients and their insurance information. At this point, it is worth mentioning that we explicitly introduce data heterogeneity and schema mismatches as potential sources of error in DA. The idea behind introducing those differences has been to investigate DEATHSTAR's capabilities to aid users in detecting potential malfunctions in the code. In our case, both relations about the relevant patient information have different names (*patients* on *Hospital A, patient_info* on *Hospital B*) and offer varying additional attributes. Participants are expected to identify these differences and adjust their code accordingly to pass the evaluation.

#### 3.1.2 User task description and survey

All participants were provided with a task description document to implement the DA code for this use case with the programming language Python and the query language SQL^16^. The programming and query language has been selected arbitrarily as our concept leverages programming language-agnostic containers. After a short oral tutorial explaining the interaction with DEATHSTAR, participants were asked to develop the analysis code for the scenario mentioned above. Alongside this main task, users were encouraged to explore the DEATHSTAR's features and functionalities. However, no further guidance or hints have been provided regarding possible issues during the development and the participants are unrestricted in how they fulfill the task. Especially, the intentionally introduced mismatch problem needs to be identified by the participants only with the help of DEATHSTAR. After the development was completed, we asked each participant to submit the code. The submitted code was then distributed and executed within the actual infrastructure. We also aimed to assess the quality of our solution from the users' perspective. Therefore, we conducted a survey upon completion of the use case implementation. The survey consisted of three parts and was conducted via an anonymous online questionnaire. The questionnaire is based on the SUS as a metric to measure the usability of a system (18, 40). The SUS consists of ten questions that are answered on a scale ranging from 1 (Strongly Disagree) to 5 (Strongly Agree) (40). From the answers to these questions, a score is calculated that ranges from 0 to 100 and indicates the system's usability, with 100 being the best reachable score (40). The final part of the evaluation consisted of six custom questions regarding the comprehensiveness and usefulness of DEATHSTAR, using the same scale as the SUS.

In total, the evaluation involved 29 participants^17^ from diverse backgrounds, such as researchers, developers, and those with experience in DA algorithm development. The evaluation sessions lasted 30–60 min on average, and the study was completed within one month. Of the participants, 11 (37.9%) reported prior experience with DA, while 18 (62.1%) stated having no prior experience. Of the 29 code submissions, 28 were executed successfully (96.6%) in the real ecosystem. All participants found the intentionally introduced schema mismatch at the two data providers and adjusted their code accordingly. However, one submission failed to establish a connection to the database since a connection parameter had been misconfigured (non-compliance with Requirement A). We have received 28 survey submissions—one submission was invalid. Based on these, we calculated the SUS according to Brooke (40). Overall, we reached a SUS score of 88.3, indicating a high level of usability. Moreover, the question, stating “*The playground solves the problem of [Smoke] testing distributed analysis algorithms”*, has an average of 4.11. Tables 2, 3 provide an overview of the user ratings. Additionally, the supplemental material^18^ provide the raw data and scripts to calculate the ratings.

**Table 2:** Average (Avg) and standard deviation (SD) per statement of the System Usability Scale (SUS) (*n* = 28).

**Question**	**Avg**	**SD**
I think that I would like to use the Playground frequently	4.21	±0.79
I found the Playground unnecessarily complex	1.43	±0.50
I thought the Playground was easy to use	4.57	±0.69
I think that I would need the support of a technical person to be able to use the Playground	1.57	±0.84
I found that the various functions in the Playground were well integrated	4.64	±0.56
I thought that there was too much inconsistency in the Playground	1.14	±0.36
I would imagine that most people would learn to use the Playground very quickly	4.46	±0.74
I found the Playground very awkward to use	1.79	±1.10
I felt very confident using the Playground	4.54	±0.58
I needed to learn a lot of things before I could get going with the Playground	1.18	±0.48

Each question could be answered on a scale from 1 (strongly disagree) to 5 (strongly agree).

**Table 3:** Average (Avg) and standard deviation (SD) per question regarding the Playground's comprehensiveness and usefulness (*n* = 28).

**Question**	**Avg**	**SD**
The Playground offers the relevant tools needed to test distributed analysis algorithms	4.50	±0.75
The schema information provided in the Playground offers all the needed information to develop an analysis task on the described data before its actual execution/deployment	4.54	±0.69
The Playground facilitates access to the schema information, which is usually sealed within the institution	4.82	±0.39
Using the Playground improves the development process—compared to deploying the analysis algorithms without the Playground	4.50	±0.75
The Playground helps with discovering possible problems in the execution, like differences in data schemas between Stations, before the execution	4.64	±0.73
The Playground solves the problem of testing distributed analysis algorithms	4.11	±0.79

Each question could be answered on a scale from 1 (strongly disagree) to 5 (strongly agree).

### 3.2 Real-world use cases

In order to showcase the adaptability and flexibility of our approach, we intend to technically evaluate it further by replicating three real-world application scenarios with more complex data structures, schemas, and data types (see Figure 4). We aim to collect performance benchmarks of DEATHSTAR, assessing its suitability for a range of scenarios with varying complexity levels of the analyses involved, usage of data instances, and (simulated) data sources. We further demonstrate the compatibility of our PoC to various underlying hardware options and perform the Smoke Tests using the CPU or the GPU. The selected use cases were previously conducted by Mou et al. and Welten et al. (13, 14). We refer to these cited references for further details about the DA experiments.

**ISIC-GEN (Summary: 10 synthetic data instances per source, three data sources, GPU only)**. The open-source dataset used for the skin lesion analysis is sourced from the ISIC^19^ and comprises image and patient metadata. Mou et al. distributed this data across three institutions in a real DA setting and conducted an experiment. In our scenario, we aim to re-model the data provision. However, this use case presents a challenge as we need to model two interlinked data sources for each data holder: A Fast Healthcare Interoperability Resource (FHIR)^20^ server for patient data and an object storage system for the skin images (as shown in Figure 4). We first developed the plugin for the FHIR server instance, and, secondly, we modeled a basic file dump to store image data. Finally, we need plugins for each modeled data type. We have decided to create plugins that generate random data, including random strings or integers, datatypes according to the FHIR standard, and even images with no semantics. Our plugins support the FHIR resource types Patient, Media, and ImagingStudy required in this use case, which are randomly filled. The chosen data type for dermoscopic images is jpeg, as it matches the format of the original images. For the jpeg-plugin, we obtained 70 placeholder images from an external service used for websites^21^. After the plugin is instantiated, these images are stored in the file dump mentioned earlier. Revisiting our main objective, we strive to offer a concept that enables Smoke Testing of algorithms. Therefore, we consider the synthetic data instances as placeholders that can be queried and processed to test the analysis, but it is not intended for producing plausible analysis results.

**ISIC-SAMPLE (Summary: 8,444 sample data instances, one data source, GPU only)**. To demonstrate that DEATHSTAR is capable of managing real) sample data and custom data sources, we replicated the ISIC-GEN use case using actual plausible sample data obtained from the ISIC repository mentioned earlier. To achieve this, we set up an external data source similar to the real setting by Mou et al. in a network accessible from DEATHSTAR's host machine instead of using our provided mechanism for data source replication.

**BC (Summary: 539 sample data instances, six data sources, CPU only)**. We conducted another use case with real data samples about BC characteristics, following a similar approach as in the previous use case. In their work, Welten et al. distributed CSV data across six institutions in a real DA setting and conducted a DA experiment on this BC dataset. We set up external storage for the CSV data, which is accessible to DEATHSTAR.

After re-modeling the required data sources, we need to develop the analysis code with DEATHSTAR. For the ISIC use cases, we developed the same image classification model, which classifies the images into benign and malign. In contrast, for BC, we implement code that trains a logistic regression model to predict BC. We implemented the analyses according to both executions paradigms, i.e., one IIL and two FL versions. Note that, regarding the FL paradigm, we implemented one fully parallelized version (original version) and one version, called FL-INC, which executes at most one analysis simultaneously. In other words, FL-INC performs IIL but updates the analysis results at the end of the round. At this point, we have provided all necessities to perform Smoke Tests on each use case. We choose three, one, and six instances for each respective scenario (as shown in Figure 4) and start the simulation. Once we successfully executed the code in the simulated environment, indicating a successful Smoke Test, we ran the DA algorithms in the PADME platform to evaluate their operability in a real-world setting. We state that all executions were as expected and successful.

## 4 Discussion

The outcomes of our first evaluation (see Section 3.1) show the effectiveness of our criteria. We observed that almost all executions of the participant's algorithms were successful. Overall, the high number of successful executions shows that our solution can indeed provide Smoke Testing capabilities for DA. The outcomes of our survey further reinforce this claim: The participants rated DEATHSTAR positively and acknowledged that it effectively “*solves the problem of [Smoke] Testing DA algorithms”* and “*offers the relevant tools needed to [Smoke] Test”* (Table 3). Beyond the results about the effectiveness, the accompanying user survey demonstrates that our realization was well-received by our study group. This result is also reflected in the SUS score of 88.3 (Table 2), placing our realization clearly above the mean score of 68 (41). Moreover, according to Bangor et al. this score can be described with an adjective rating of “excellent”, placing it in the highest out of four quartiles (18). When we investigate the cohorts, including participants with and without prior experience, only a small difference in the SUS score is visible: Participants with a background in DA rated our concept with a score of 86.6 compared to a rating of 89.8 by the unfamiliar users. All participants have been able to “*discover possible problems in the execution, like differences in data schemas, before the execution”* with DEATHSTAR. Additionally, the participants appreciated the ability to employ a trial-and-error approach during development.

In the second and more technical evaluation, we assessed the flexibility of our approach by applying it to real-world use cases. We have been able to use DEATHSTAR for generating data and creating complex, interlinked data sources, indicating that its concept is capable of working with very distinct settings such as structured data, images or textual data. We would like to emphasize that the same code used for ISIC-GEN also worked for ISIC-SAMPLE, indicating that our approach involving synthetic data was able to successfully replicate data sources used in the real-world use case (ISIC-SAMPLE). During our technical evaluation, we additionally measured the duration of each Smoke Test (i.e., simulation). Note that each analysis code has to be containerized before the simulation. As this factor might also count as part of the Smoke Test, we also measured the image-building time (see Table 4). All builds have been executed without pulling the overarching Python image for the analysis container, and the needed dependencies have been downloaded with a connection speed of 900 MBits. In the scope of this technical evaluation, DEATHSTAR has been deployed on a server with 4 × 3.60 GHz CPU, 128 GB RAM, and a TITAN XP GPU.

**Table 4:** Each row represents the measured duration for the building times of the images, the time for one single provider, and the time for a complete Smoke Test.

**Use case**	**Build**	**One Data** **Source**	**Smoke Test** **IIL**	**Smoke Test** **FL**	**Smoke Test** **FL-INC**
User-study	23 s	6 s	12 s	15 s	17 s
ISIC-GEN	1 m 39 s	24 s	1 m 6 s	56 s	1 m 15 s
ISIC-SAMPLE	1 m 39 s	4 m 31 s	–	–	–
BC	6 m 6 s	48 s	4 m 33 s	11 m 53 s	4 m 51 s

Note that the ISIC-SAMPLE use case has only been conducted on one data source.

Based on these measured times, we can derive three factors that influence the Smoke Tests:

1. **Analysis complexity:** While the Smoke Test of the User-Study case terminates almost immediately, the more complex data analyses ISIC-GEN, ISIC-SAMPLE, and BC need more time since these involve ML model training, whose duration is usually influenced by the number of epochs or the complexity of the to be trained model itself. Additionally, we can identify another effect, which is the number of required dependencies used for the analyses. Due to our design based on containerization, DEATHSTAR builds an image for each analysis. Hence, each dependency has to be included. This results in the BC analysis image needing more time to be built than the ISIC images since the BC image covers more packages. However, note that many packages can be cached once an image has been built. This caching reduces the build times to >2 s.
2. **Dataset size:** Similar to the analysis complexity, the number of used data instances for the Smoke Tests influence its duration. While the analysis code for User-Study and ISIC-GEN only processes 10 instances per provider (fastest), BC processes 539 instances, and the ISIC-SAMPLE analysis queried 8,444 images (slowest).
3. **Number of simulated data sources:** The more providers are involved in the Smoke Test, the longer the duration. This can be explicitly seen in ISIC-GEN and BC, where we involved three and six providers, respectively. Thus, the simulation duration is directly influenced by a factor proportional to the number of data sources.

Regarding the three characteristics of Smoke Testing, we can derive the following connections and conclusions from our evaluation results. By simulating the analyses, DEATHSTAR can identify potential issues and problems in the algorithm's functionality without having to perform an exhaustive and extensive test. This contributes to the “shallow” characteristic, and the high number of error-free executions underpin the effectiveness of our criteria. Regarding the “fast” characteristic, we face a trade-off between the duration of the Smoke Tests and three factors that influence the simulation, as discussed above. At this point, we argue that the Smoke Test can be optimized, for example, by using fewer data sources (e.g., in the case of homogeneous data sources) or fewer data instances. For example, the ISIC-SAMPLE use case also works using a fraction of the 8,444 images, which might reduce the Smoke Test duration significantly (see ISIC-GEN). Furthermore, there is potential for improvement in implementing the FL paradigm. While executing the fully parallelized version (FL) in the BC use case, we encountered a slowdown of the Smoke Test due to the increased loads produced by the parallel execution. An alternative that circumvents the concurrency issues and therefore offers faster Smoke Testing could be FL-INC, which exhibits similar performance to IIL. Finally, regarding the “automated” characteristic, we found that through our simulation-based approach, we enable a fully automated Smoke Test with minimal manual intervention. Each Smoke Testing criterion mentioned above is automatically validated by our Simulation Engine, contributing to a seamless use of DEATHSTAR, partially shown by our survey results.

### 4.1 Threats to validity

Some limitations have become apparent that can be attributed to our design decisions. While DEATHSTAR fully automates the Smoke Tests, some prior efforts still have to be devoted to collecting the schema information from each data source, which could pose a bottleneck. This especially holds for the creation of plugins and the data re-modeling in case sample data is unavailable for Smoke Testing. Although we included the aspect of reusability in our design decisions (“mix-and-match”) and our already developed assets can be used as foundations, the aspect of re-modeling data sources might still be a time-consuming and error-prone factor. Since our main objective has been the definition of Smoke Testing criteria for DA analyses, we mainly focused on the effectiveness of our criteria. Hence, our evaluation does not cover the aspect of data re-modeling, and this question remains open. The second threat is our implementation as such. Our simulation might produce an overhead in the Smoke Testing strategy that might validate additional requirements implicitly, which influences the effectiveness of our approach. The defined criteria can be tested through another approach beyond simulation that tests each criterion individually. This threat has also been analogously stated in work by Cannavacciuolo and Mariani (17). We have chosen a *testing-through-simulation* approach to comply with the “automated” characteristic and the iterative manner of DA analyses. Hence, we argue that our approach provides the flexibility to master the sheer amount of data source technologies, schemas, or analysis types. Validating each criterion separately for each DA scenario might impede this flexibility. However, the benchmarking of our concept against other similar approaches remains open.

In summary, in this work, we addressed the issue of lacking Smoke Testing criteria for the validation of DA code. We have pointed out that insufficiently tested analysis code is susceptible to SPOFs, which causes a complicated and time-consuming development process due to the inherently decentralized nature of DA infrastructures and the dependence on the data providers during development. In order to tackle this issue, we propose six criteria that must be guaranteed to ensure the operability of the analysis code, representing a successful Smoke Test (RQ1). Based on these criteria, we developed a PoC, called DEATHSTAR, that locally performs Smoke Testing on DA code following a *testing-through-simulation* approach by simulating an entire DA experiment (RQ2). Since the application of Smoke Testing to data analyses is dependent on the availability of sufficient sample data, we leveraged a flexible and adaptable plugin system, which allows the semi-automated creation of synthetic test data, which can be used for Smoke Testing (RQ2.1 & RQ2.2). Hence, we developed a solution that allows users to develop iteratively (i.e., trial-and-error) and (Smoke) test their analysis code by simulating its execution on re-modeled data sources. We evaluated DEATHSTAR in a two-folded evaluation. First, we conducted a User-Study with 29 participants to evaluate the effectiveness of our criteria. We found that 96.6% of all developed DA analyses that were initially Smoke Tested could be successfully executed in a real DA environment. Furthermore, our accompanying survey resulted in a SUS score of 88.3, giving DEATHSTAR an “excellent” usability rating. Secondly, we applied DEATHSTAR to three real-world use cases in the scope of a technical evaluation. The technical results of our evaluation show that our concept is flexible enough to serve for different use cases and complies with the three characteristics of Smoke Testing: Shallow, Fast, and Automatic. In conclusion, within the scope of our work, the contribution of our PoC fuels research by reducing obstacles in conducting DA studies.

## Data availability statement

The datasets presented in this study can be found in online repositories. The names of the repository/repositories and accession number(s) can be found in the article/supplementary material.

## Author contributions

SWel: Conceptualization, Formal analysis, Methodology, Validation, Writing – original draft, Writing – review & editing. SWeb: Conceptualization, Methodology, Software, Validation, Writing – original draft, Writing – review & editing. AH: Software, Validation, Writing – review & editing. OB: Supervision, Writing – review & editing. SD: Supervision, Writing – review & editing.

## Footnotes

^1^GDPR: www.gdpr-info.eu.

^2^ https://www.europarl.europa.eu/RegData/etudes/STUD/2022/740054/IPOL_STU(2022)740054_EN.pdf

^3^The code of the PoC is available as Open Source, including screenshots, a screencast, examples, the developed data schema, and explanations at: https://github.com/PADME-PHT/playground.

^4^Further DS studies are available at: www.datashield.org/about/publications.

^5^Open Container Initiative: https://opencontainers.org.

^6^Terse RDF Triple Language: www.w3.org/TR/turtle/.

^7^Schema and examples: https://github.com/PADME-PHT/playground.

^8^Plugins: https://github.com/PADME-PHT/playground/tree/main/src/backend/src/lib/data-generator/plugins.

^9^Docker: www.docker.com.

^10^PyTorch: https://pytorch.org/.

^11^Scikit-Learn: https://scikit-learn.org.

^12^Python: https://www.python.org/.

^13^R: https://www.r-project.org/.

^14^Detailed results and resources for replicating the evaluations to are provided at: https://github.com/PADME-PHT/playground/tree/main/evaluation.

^15^For a video demonstration of the use case task see https://github.com/PADME-PHT/playground.

^16^PostgreSQL Syntax: https://www.postgresql.org.

^17^Raw data and details about the evaluation: https://github.com/PADME-PHT/playground/tree/main/evaluation/user_study.

^18^Supplemental material can be found here: https://github.com/PADME-PHT/playground.

^19^ISIC Challenge: www.isic-archive.com.

^20^FHIR standard: https://hl7.org/fhir.

^21^LoremFlickr CC): www.loremflickr.com.
